# Malawer limb salvage surgery for the treatment of scapular chondrosarcoma

**DOI:** 10.1186/1477-7819-12-196

**Published:** 2014-06-30

**Authors:** Fei Chang, Guang-Yao Liu, Qiao Zhang, Gang Lin, Hong Huang, De-Sheng Duan, Jin-Cheng Wang

**Affiliations:** 1 Department of Orthopedic Surgery, the second hospital of Jilin University, Changchun, China; 2 Department of Orthopedic Surgery, China-Japan Union Hospital of Jilin University, Changchun, China

**Keywords:** Chondrosarcoma, Scapula, Limb salvage surgery

## Abstract

Chondrosarcoma is a common malignant bone tumor, which accounts for 20% of all malignant bone tumors. It often occurs in the long bones, but the incidence of scapular chondrosarcoma is rare. Here, we describe a case of a large chondrosarcoma occurring in the scapula which was treated with Malawer limb salvage surgery. The patient retained considerable limb function after complete removal of the tumor tissue as assessed at the follow-up visit two years and ten months following surgery.

## Background

Chondrosarcoma is a malignant bone tumor and it is the second highest cause of morbidity among adult bone tumors. It accounts for 20% of all malignant bone tumors. Chondrosarcomas constitute a heterogeneous group of neoplasms that share the common feature of producing the cartilage matrix [1]. Generally, chondrosarcoma is intramedullary, and the fundamental tumor tissue is the new cartilage tissue, sometimes with ossification, calcification and mucoid degeneration. The tumor always has a primary origin, but a few secondary cases have also been reported [2]. The main symptoms are pain and swelling. As radiology and chemotherapy are usually ineffective, the treatment most often used is surgical resection. If a complete surgical resection is not performed, the tumors reappear in the lesions and, rarely, lead to metastasis to distant sites. The prognosis depends largely on the location of the original lesion, obtaining negative sections and the histological degree of the tumor [3-6].

Chondrosarcoma does not usually develop in the osteoid tissue. It usually occurs in cartilage of the pelvis, femur, tibia and humerus. Scapular chondrosarcoma is rare, accounting for about 5 to 7% of all reported cases of chondrosarcoma [7,8]. Until the 1970s, amputation was the preferred treatment for severe cases of malignant tumors around the scapula. Since then, an increasing number of patients have adopted the limb salvage treatment as the first choice, and in most cases, the outcomes have been good [9]. Although there have been many reports of the use of limb salvage treatment, the tumor was not large and there was no damage to the soft tissue, nerves and blood vessels around the tumor in most studies. To our knowledge, there are few reports on the use of limb salvage treatment for patients with extremely large tumors. Also, the relevant risks and the efficacy of such therapy for the treatment of large tumors are unknown.

In this study, we present a case of a large chondrosarcoma in the scapular area. Based on the preoperative evaluation of the surrounding soft tissue and the reconstruction design, the patient was treated with Malawer limb salvage surgery. No complications were reported postoperatively and the patient retained limb function.

## Case presentation

**Figure 1:**
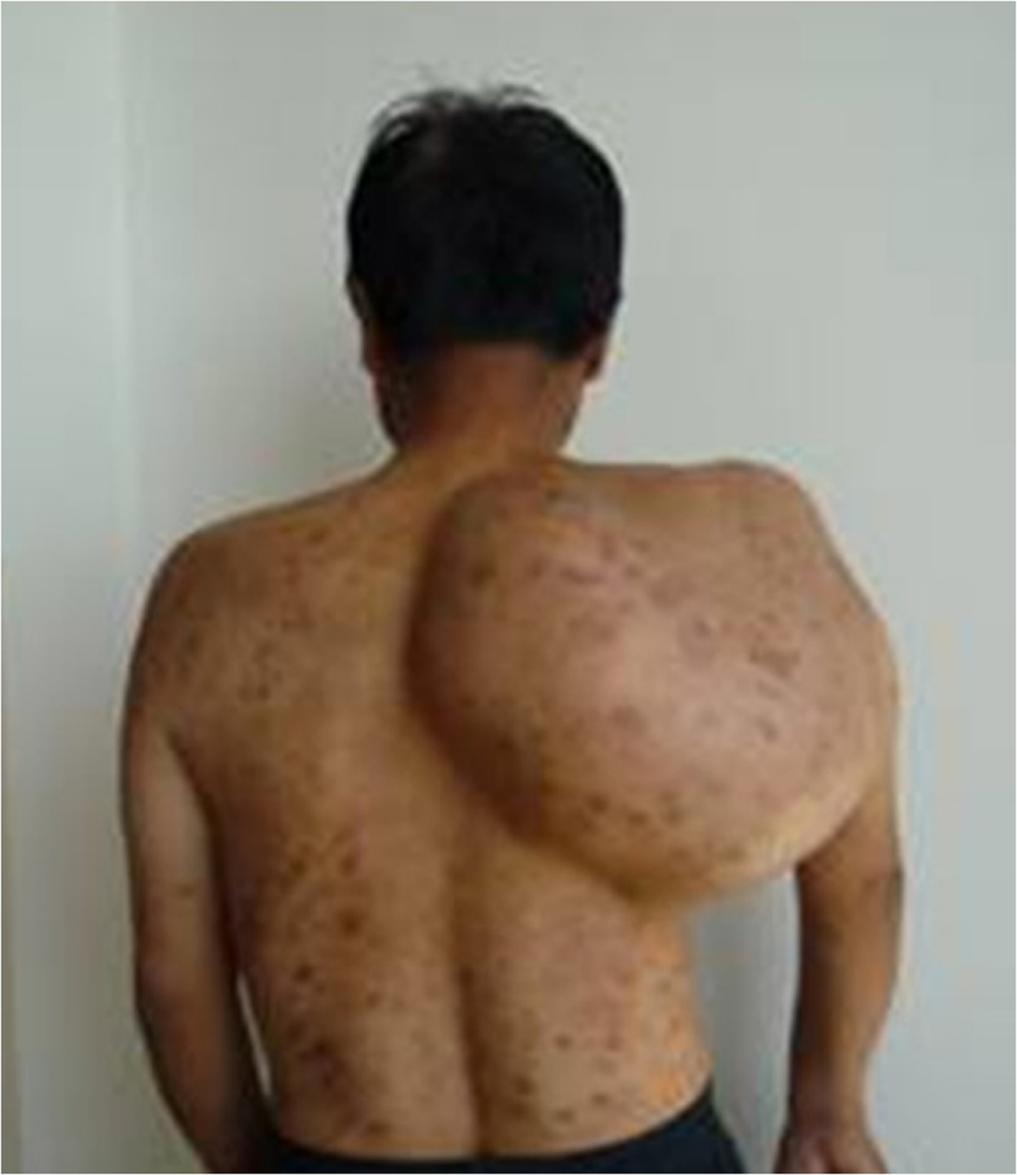
**The photograph taken preoperatively showing the tumor in the right scapula.** The size of the mass was about 38 × 33 × 30 cm.

**Figure 2:**
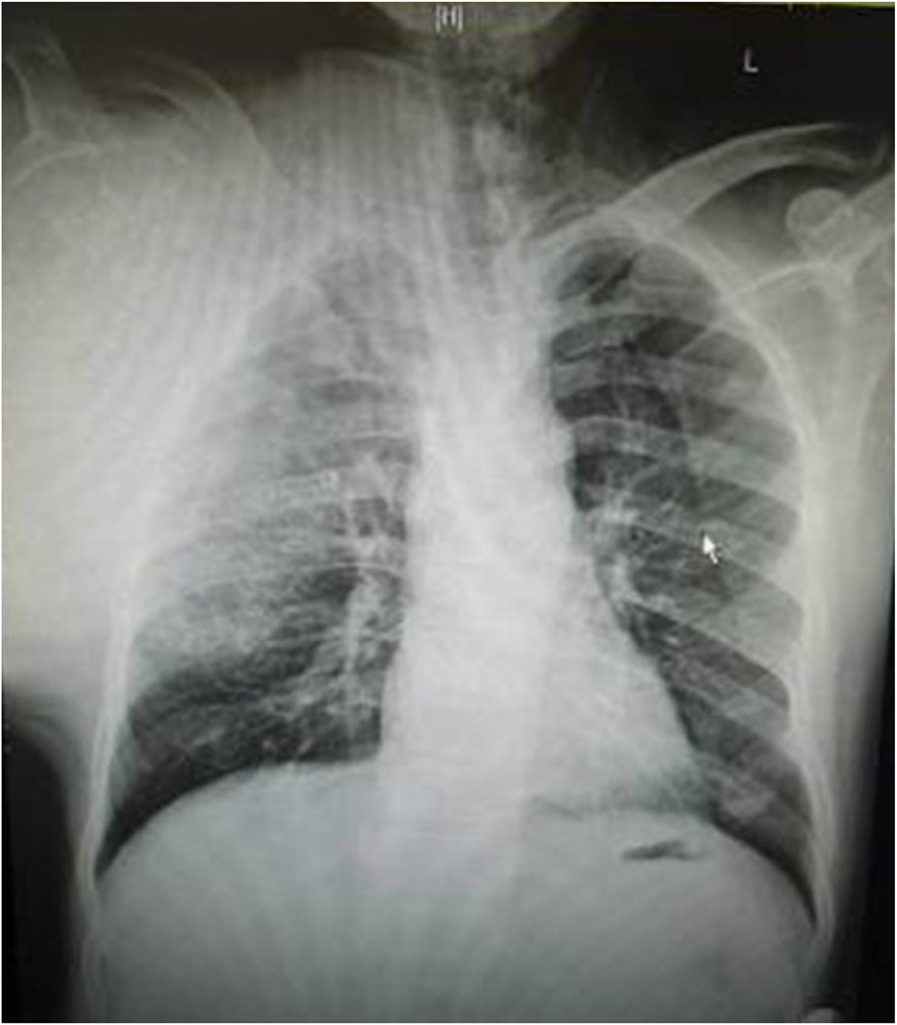
The radiograph of the right shoulder showing the tumor in the right scapula before surgery.

**Figure 3:**
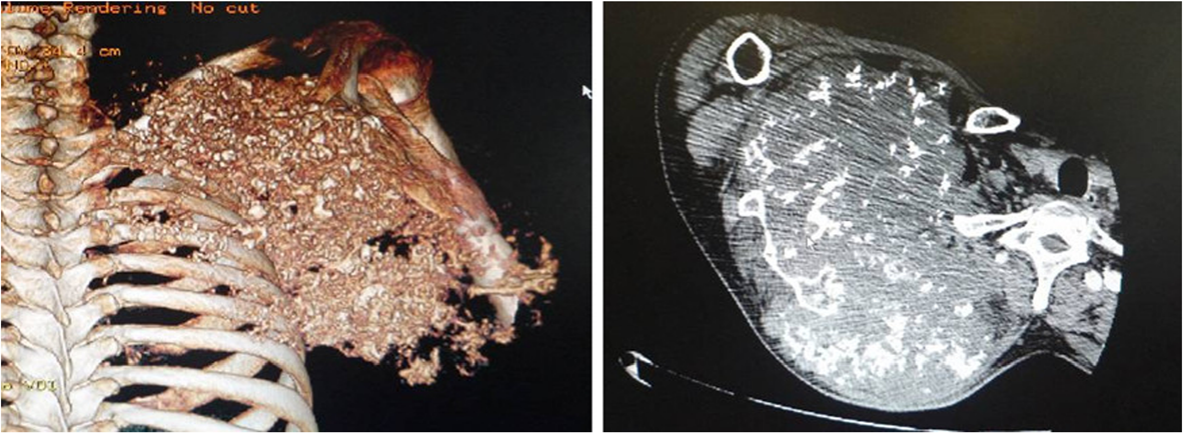
The computer tomography (CT) image of the right shoulder showing the tumor in the right scapula before surgery.

**Figure 4:**
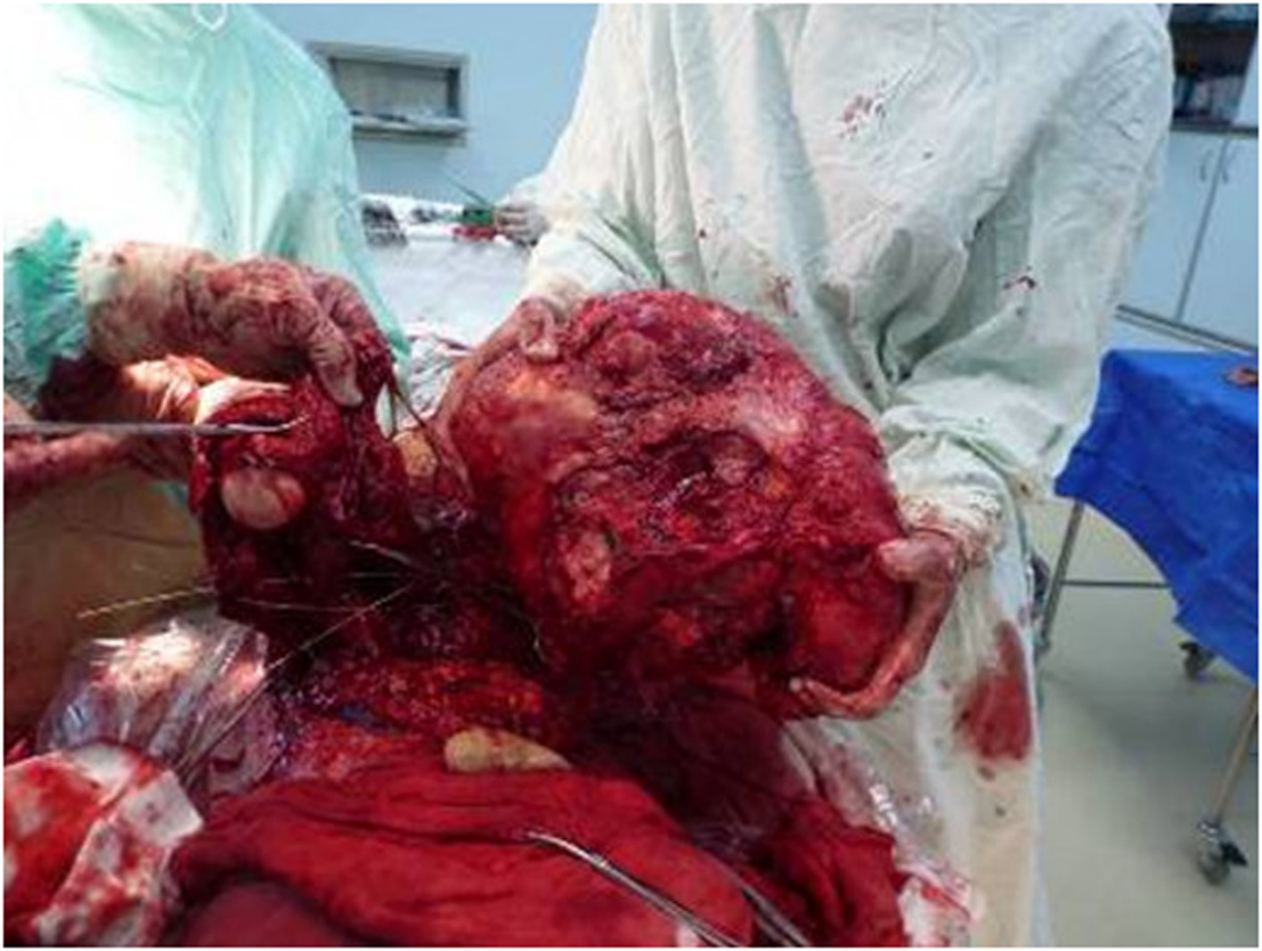
The photograph showing that the tumor had been completely removed during the operation.

**Figure 5:**
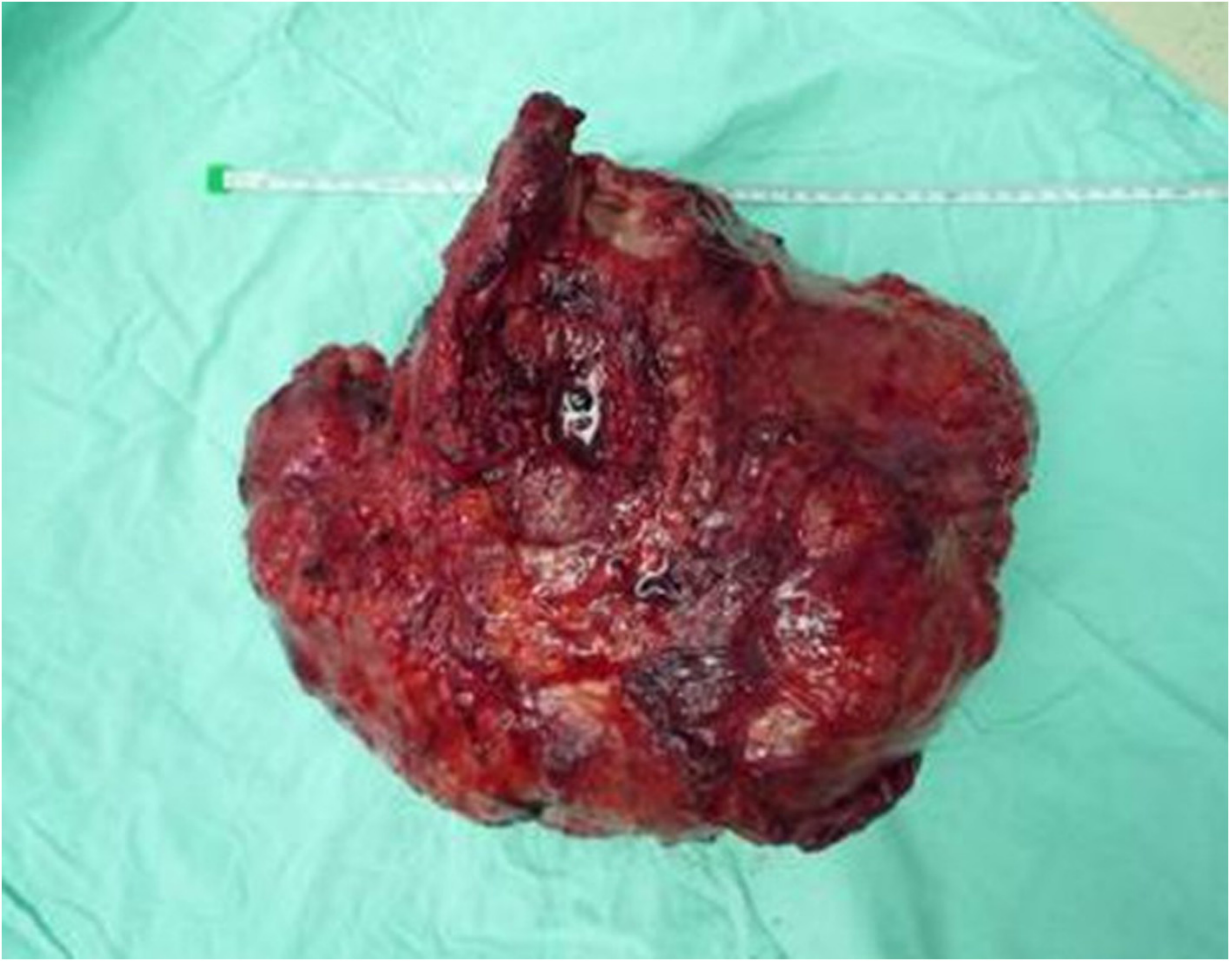
The photograph showing the removed tumor.

**Figure 6:**
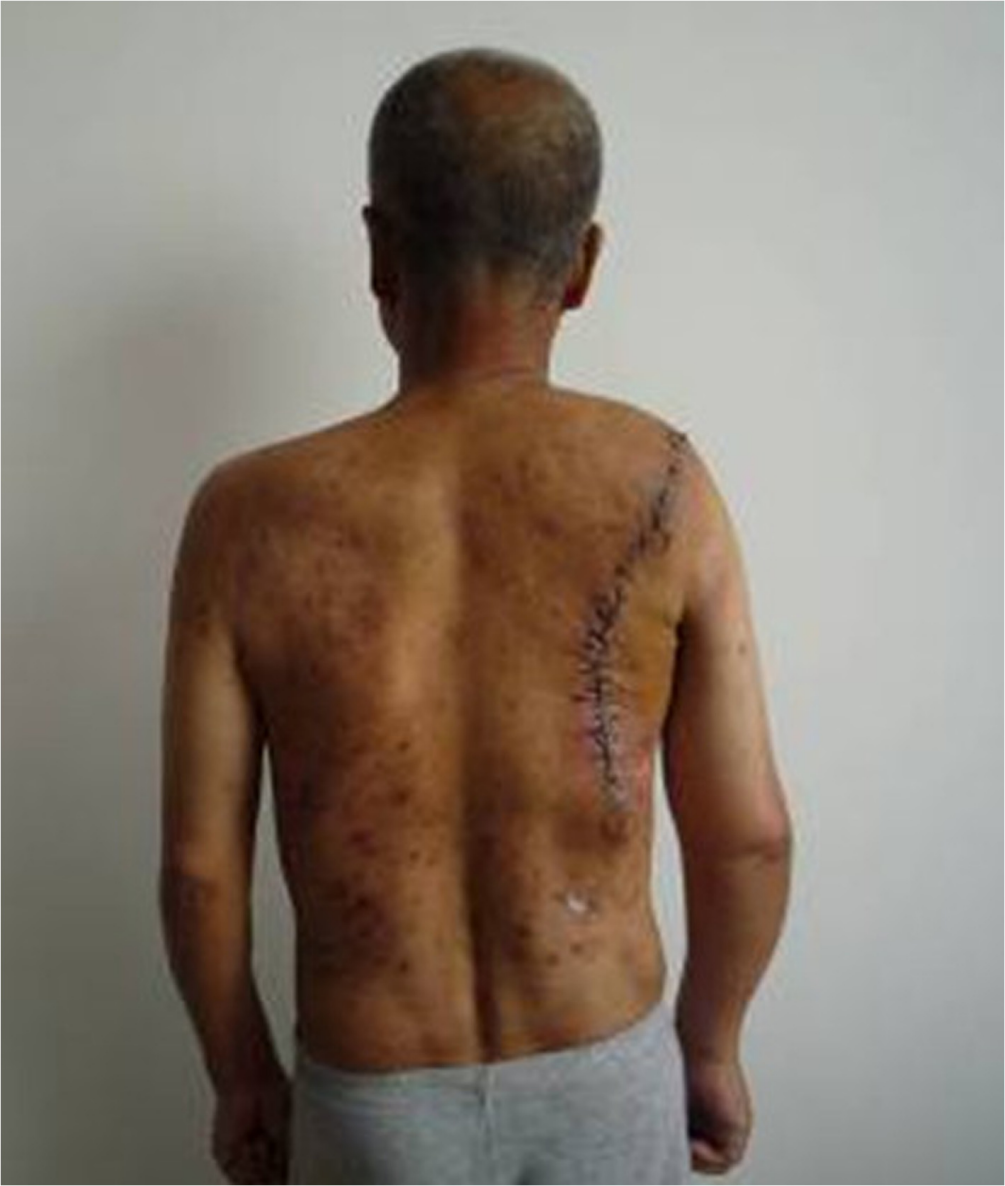
The photograph showing that the right shoulder of the patient is nearly recovered and has a normal postoperative appearance.

**Figure 7:**
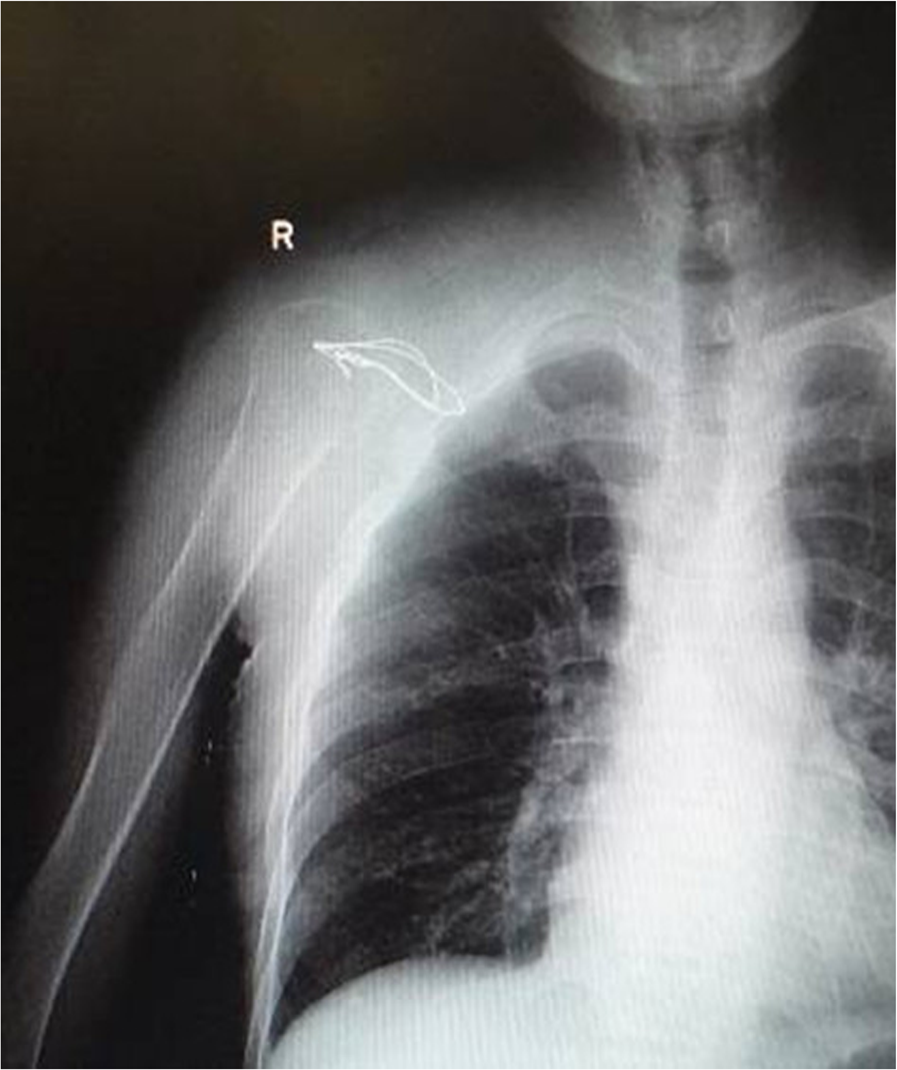
The postoperative radiograph of the right shoulder.

**Figure 8:**
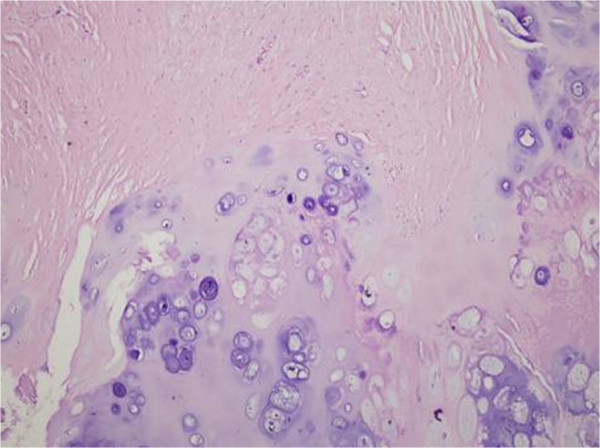
The postoperative pathological examination of the tumor tissues.

The patient was in compliance with the Declaration of Helsinki. A 59-year-old farmer with a personal history of a lump on his right scapula for the past five years was admitted to hospital. In two years prior to the first hospital visit, the lump had grown significantly and had restricted the activity of the right shoulder. The patient did not have a family history of tumors.Physical examination indicated no apparent spread to the superficial lymph nodes. There was a huge, irregular lump in the region of the right shoulder, with the superior border reaching the acromion, the left border reaching to 1 cm from the spinal column, the inferior border reaching the seventh rib, and the lateral border reaching the midaxillary line. The lump was nodular, hard and caused a pressing pain. There was an obscure boundary between the tumor and the surrounding tissues, the skin around the tumor was red but there was no venous engorgement or vascular pulsation (Figure 1). Fifty degree abduction of the right shoulder joint, 0° adduction, 30° flexion, 40° extension and 10° external and internal rotation were observed. The pulsation of the right ulnar artery and the right radial artery were good. Myodynamia of the main right upper extremities were as follows: supraspinatus at level III, deltoid at level IV, muscles of biceps brachii at level IV, extensor carpi at level IV, flexor digitorum profundus at level IV and abductor digiti quinti at level IV.The results of the vascular ultrasound of the right clavicle and the axilla were as follows: the axillary artery was visible in front of the tumor with smoothly flowing blood; there was an obvious boundary between the subclavian artery and the tumor with smoothly flowing blood. The results of the X-ray (Figure 2) and computer tomography (CT) scan (Figure 3) were as follows: a large high-density mass was seen in the right scapula and the surrounding tissue with multifocal calcification. As can be seen in the X-ray and the CT scan, the entire scapula was involved. In addition, the scapula showed osteolytic destruction without normal structure and the remaining scapula was embedded in the tumor tissue. There were no abnormalities adjacent to the ribs, humerus and thoracic vertebra. The isotope bone scan showed the concentration of radioactivity in the right scapular area with abnormal mineral metabolism in the bones. The biopsy indicated grade II (out of III) chondrosarcoma. The diagnosis was as follows: chondrosarcoma in right scapular, Malawer S1 S2 district, Enniking II B district, grade II chondrosarcoma.Two weeks after hospitalization, the patient was treated with the Malawer III limb-reserving surgery under general anesthesia. The levator scapulae, rhomboid, supraspinatus, infraspinatus and teres muscles along the medial walls of the scapula were stripped and resected in an order based on the principles of wide resection. Simultaneously, the nerves and blood vessels that enter the tumor tissue were excised. The tumor was widely adherent to the surrounding soft tissue. Also, its relationship with the axillary artery and the subclavian artery was obscure. It was difficult to directly turn the rib. Under these circumstances, the incision was extended, the clavicle was resected and then the teres was moved downward. There was quite a close relationship between the subclavian artery, the axillary artery and the tumor, but there was some separation with soft tissue envelope. The serratus anterior was partly cut down to completely expose the tumor (Figure 4 and Figure 5). The scapular was entirely embedded in the tumor tissue, hence it was removed while the tumor was resected. The size of the tumor was 33 × 28 × 25 cm. There was no involvement of the proximal humerus and the thoracic vertebrae. Then, the lesions and the surrounding tissue were obtained for pathological analysis. As the pathology results of the surrounding tissue were negative, the humerus was attached to the second rib with a steel wire, then the supraspinatus, infraspinatus, teres minor and the rhomboid muscles were sutured. The trapezius, ectopectoralis and deltoid were sutured together. The brachialis, musculus biceps brachii and musculus triceps brachii were sutured together and fixed to the first rib. The brachycephaly of musculus biceps brachii was fixed to the second rib to ensure the balance of the shoulder joint (Figure 6). The patient was required to maintain his elbow in a flexed position with a bandage for three weeks. The postoperative X-ray showed that no tumor tissue remained (Figure 7). On the first postoperative day, the myodynamia and sensation of the major muscles under the right shoulder were the same as prior to the operation. On the third postoperative day, the patient exercised his hand and wrist functions with a patient controlled epidural analgesia. At postoperative week three, partly loading functional training was performed and at the sixth week, fully loading functional training was performed. The stitches were removed two weeks postoperatively without any complications. The pathology report was almost the same as that prior to the operation (Figure 8). The MSTS (Musculoskeletal Tumor Society Score) score was 22 one year after the surgery. Postoperative radiography at 3, 6 and 18 months detected no tumor recurrence. There was no evidence of metastasis at the follow-up end point.

## Discussion

### Surgery

Surgery is the leading treatment for chondrosarcoma. In the past, amputation was always adopted as the treatment for malignant tumors in the scapular area, but this destroyed the balance of the body, negatively impacted the appearance and functions of the body and caused emotional harm to the patients. In recent years, there have been an increasing number of studies in oncology and biological therapy for the treatment of tumors as well as the development of reconstruction science when sufficient tissue is not available. Therefore, 90 to 95% of the malignant tumors can now be safely treated with limb salvage surgery [10]. Statistics for clinical evidence-based medicine related to chondrosarcoma indicate there are no significant differences in negative resectioning between amputation and limb salvage surgery as well as improvement in disease-free survival period and decrease in recurrence rates [11-14]. Although the grade of the tumor affects the prognosis, there are studies that suggest that factors, such as age, gender, tumor volume, classification and resection method do not significantly influence the recovery of the limb function [15].

Shoulder girdle resections were classified according to the classification system proposed by Malawer and colleagues in 1991 [16]. It is based on anatomic location, extent of the tumor, and tumor grade. In the classification scheme, resection types are numbered I to VI (Table 1). In general, types I to III resections are intra-articular. They are used to manage benign, low-grade malignancies or metastatic lesions. Types IV to VI resections are extra-articular and are used to manage high-grade primary sarcomas of the shoulder girdle.

**Table 1:** Surgical classification system of limb-sparing resections of the shoulder girdle

	
Type I	Intra-articular proximal humeral resection
Type II	Partial scapulectomy
Type III	Intra-articular total scapulectomy
Type IV	Extra-articular scapular and humeral head resection
Type V	Extra-articular humeral and glenoid resection
Type VI	Extra-articular humeral and total scapular resection

For the case presented in this study, the tumor in the scapular area was adjacent to important nerves and blood vessels, and also involved various surrounding soft tissues. Therefore, the requirement to expose the tumor, excessive bleeding and avoiding nervous dysfunction made the surgery difficult to perform. Moreover, there are many complications that may follow function reconstruction procedures. However, with reasonable reservation and reconstruction of the soft tissue and bone structure, the upper limb function can be preserved. In this case, based on preoperative radiography, the patient’s diagnosis was IIB phase of a G1T2MO tumor according to the AJCC (American Joint Committee on Cancer) staging system, without apparent involvement of nerves and blood vessels, nearby chest wall or proximal humerus. Due to the low invasive ability of chondrosarcoma, we believed that it was possible to perform the surgical resection with wide margins and preserve the soft tissue in order to preserve the proximal humerus that provided a pivot for the elbow, wrist and hand. The ultimate goal was to improve the quality of life if complete resection of the tumor could be performed.

### Function reconstruction

Limb-sparing surgical procedures have replaced the forequarter amputation as the surgical treatment of choice for tumors in and around the shoulder girdle. Bickels *et al*. reported 134 patients who underwent a limb-sparing resection, and after a minimum of two years follow- up, local tumor control, associated with good functional outcomes, was achieved in the majority of patients [17]. There are various methods for the reconstruction of the scapula, such as allogeneic bone or joint transplant, inactive bone replant, artificial prosthesis replacement, joint fusion, non-bone reconstruction, and so on. Each method has its own advantages and disadvantages. Allogeneic bone or joint has the same bone shape and strength and the knitting meets the criteria of biomechanics; however, there are associated problems, such as rejection reaction and delayed recovery [18]. Inactive segmental bone matches the host bone in terms of the immunology and the structure, bone conduction and bone induction. The major disadvantages associated with the use of inactive segmental bone are the recurrence of the tumor and increasing incidence of fractures and infections as the strength of the inactive bone decreases. Some researchers have reported that replantation of the boiled analogous scapulae or reconstruction of microwave-devitalized tumor bone has led to good outcomes [19]. Artificial prostheses can reconstruct the structure and the dynamics of the shoulder joint, and can be instantly stabilized. The function of the shoulder joint following prosthesis depends on a number of preserved muscles. Villalobos *et al*. reported that the functional outcome of total scapula prosthesis is superior to forequarter amputation [20]. The aim of non-bone reconstruction of Malawer III is to fix the proximal humerus to the second rib or the clavicle in an appropriate manner. Humeral suspension is a popular reconstructive procedure after total scapulectomy, and the patients treated with this surgical method have acceptable shoulder function although the appearance is poor. In another study, Pritsch *et al*. found that the scapular endoprosthesis was functionally superior to humeral suspension after total scapulectomy [21].

The reconstruction of the bone and the joint depends on the reconstruction of the soft tissue regardless of the method that may be adopted. The crucial factor in limb salvage surgery is the careful evaluation of the condition of the soft tissue. If it is assessed that no available soft tissue can be preserved and there is no appropriate way of reconstruction, then limb salvage surgery will lead to a huge economic burden and emotional harm. Our principle is to save as much soft tissue as possible on the condition that the tumor is completely resected. Rotator cuff, deltoid and musculus biceps brachii are the key muscles in sustaining shoulder function; therefore, these are preserved better in order to form a triangle and to efficiently transfer the articular forces. In the present case, the chondrosarcoma was large but the soft tissue and the skin were in good condition. We estimated that there would be enough soft tissue and skin to cover the trauma after the enlarged resection. We took the activity level of the shoulder into consideration, so that the function of the shoulder may be preserved postoperatively. During the operation, our judgment was proved correct and the soft tissue was good enough to perform the limb salvage surgery. After the surgery, the patient retained shoulder function and his basic living requirements could be met even though the appearance was not good.

## Conclusions

In the present case, the tumor was large and many nearby tissues had been invaded, especially anteriorly. The tumor was very near to the axillary nerves and blood vessels. Therefore, it was very difficult to perform the limb salvage surgery and considerable risks were associated with the surgical procedure. Moreover, wide resection of tumor can lead to loss of viability of the skin and the soft tissue. All these issues need to be considered and planned carefully preoperatively. This case is a unique one and cannot provide a therapeutic guide for chondrosarcoma. However, the operation was a great success and the patient showed good recovery. This case provides an example for the treatment of giant chondrosarcomas.

## Consent

Written informed consent was obtained from the patient for the publication of this report and any accompanying images.

## Abbreviations

AJCC: American Joint Committee on Cancer; CT: computer tomography; MSTS: Musculoskeletal Tumor Society Score.

## Competing interests

The authors declare that they have no conflicts of interest concerning this article.

## Authors’ contributions

FC, GYL, QZ, DSD, JCWcarried out the operation; FC drafted the manuscript; HH carried out the histology analysis; GYL carried out the patient follow-up; JCW performed the design of the operation and coordination and helped to draft the manuscript. All authors read and approved the final manuscript.
